# Rationale and design of the B-PROOF study, a randomized controlled trial on the effect of supplemental intake of vitamin B_12 _and folic acid on fracture incidence

**DOI:** 10.1186/1471-2318-11-80

**Published:** 2011-12-02

**Authors:** Janneke P van Wijngaarden, Rosalie AM Dhonukshe-Rutten, Natasja M van Schoor, Nathalie van der Velde, Karin MA Swart, Anke W Enneman, Suzanne C van Dijk, Elske M Brouwer-Brolsma, M Carola Zillikens, Joyce BJ van Meurs, Johannes Brug, André G Uitterlinden, Paul Lips, Lisette CPGM de Groot

**Affiliations:** 1 Department of Human Nutrition, Wageningen University, P.O. Box 8129 6700 EV Wageningen, the Netherlands; 2 Department of Epidemiology and Biostatistics and the EMGO Institute for Health and Care Research, VU University medical center, Van der Boechorststraat 7, 1081 BT Amsterdam, the Netherlands; 3 Department of Internal Medicine-Section Geriatric Medicine, Erasmus MC, University Medical Centre Rotterdam, P.O. Box 2040, 3000 CA Rotterdam, the Netherlands; 4 Department of Internal Medicine, Erasmus MC, University Medical Centre Rotterdam, P.O. Box 2040, 3000 CA Rotterdam, the Netherlands; 5 Department of Endocrinology, VU University medical center, P.O. Box 7057, 1007 MB Amsterdam, the Netherlands

## Abstract

**Background:**

Osteoporosis is a major health problem, and the economic burden is expected to rise due to an increase in life expectancy throughout the world. Current observational evidence suggests that an elevated homocysteine concentration and poor vitamin B_12 _and folate status are associated with an increased fracture risk. As vitamin B_12 _and folate intake and status play a large role in homocysteine metabolism, it is hypothesized that supplementation with these B-vitamins will reduce fracture incidence in elderly people with an elevated homocysteine concentration.

**Methods/Design:**

The B-PROOF (B-Vitamins for the PRevention Of Osteoporotic Fractures) study is a randomized double-blind placebo-controlled trial. The intervention comprises a period of two years, and includes 2919 subjects, aged 65 years and older, independently living or institutionalized, with an elevated homocysteine concentration (≥ 12 μmol/L). One group receives daily a tablet with 500 μg vitamin B_12 _and 400 μg folic acid and the other group receives a placebo tablet. In both tablets 15 μg (600 IU) vitamin D is included. The primary outcome of the study is osteoporotic fractures. Measurements are performed at baseline and after two years and cover bone health i.e. bone mineral density and bone turnover markers, physical performance and physical activity including falls, nutritional intake and status, cognitive function, depression, genetics and quality of life. This large multi-center project is carried out by a consortium from the Erasmus MC (Rotterdam, the Netherlands), VUmc (Amsterdam, the Netherlands) and Wageningen University, (Wageningen, the Netherlands), the latter acting as coordinator.

**Discussion:**

To our best knowledge, the B-PROOF study is the first intervention study in which the effect of vitamin B_12 _and folic acid supplementation on osteoporotic fractures is studied in a general elderly population. We expect the first longitudinal results of the B-PROOF intervention in the second semester of 2013. The results of this intervention will provide evidence on the efficacy of vitamin B_12 _and folate supplementation in the prevention of osteoporotic fractures.

**Trial Registration:**

The B-PROOF study is registered with the Netherlands Trial (NTR NTR1333) and with ClinicalTrials.gov (NCT00696514).

## Background

Osteoporosis is a chronic, multifactorial disorder which is characterized by low bone mass and micro architectural deterioration of bone tissue [1]. Its major consequence is fractures, and especially hip fractures are associated with institutionalization and increased mortality. In 2000, approximately 9 million fractures occurred worldwide, leading to a loss of 5.8 million disability adjusted life-years (DALYs) [2]. Due to a rise in life expectancy, the economic burden of osteoporotic fractures in Europe is expected to increase substantially in the coming decades: from €36.3 billion in 2000 to €76.8 billion in 2050 [3].

Pharmacological interventions may prevent 30-60% of fractures in patients with osteoporosis [4]. However, due to the high prevalence of osteoporosis and osteoporotic fractures, attention has been shifted towards preventive lifestyle interventions, such as vitamin D and calcium supplementation and promoting physical activity. Vitamin D and calcium supplementation has been shown to decrease the incidence of hip fractures and other non-vertebral fractures by 23-26% [5]. Increased physical activity is related to higher bone mineral density (BMD), bone structure and elasticity [6,7] and is suggested to reduce the risk of hip fracture [8].

Besides those well-established factors, it has been shown that elevated homocysteine concentrations and low vitamin B_12 _status are strongly associated with lower bone mass and higher fracture risk in independent living elderly [9-11] and frail elderly [12]. Vitamin B_12 _and folate deficiencies and elevated homocysteine concentrations have been associated with lower BMD [13-18].

An elevated plasma homocysteine concentration (≥ 15 μmol/L) is prevalent in 30-50% of Dutch people older than 60 years, increases with age [19-21] and is multifactorial; age, sex and lifestyle factors, as well as environmental and genetic factors, nutritional intake of B-vitamins and hormonal factors affect homocysteine status [22]. B-vitamins play a central role in the homocysteine metabolism [23]. Treatment with vitamin B_12 _and folic acid supplements is effective in normalizing homocysteine concentrations [24,25].

Evidence of a beneficial effect of supplementation with B-vitamins on fracture incidence has been signalled in Japan in elderly hemiplegic patients following stroke [26]. However, the generalizability of these findings is limited, since a highly selective patient population with a high percentage of vitamin D deficiency and a high fracture risk was studied. Moreover, pharmacological doses of folic acid (5 mg/day) and vitamin B_12 _(1.5 mg/day) were given, which may increase the risk of adverse effects.

*In vitro *studies support the hypothesis of a beneficial effect of vitamin B12 supplementation. Vitamin B_12 _has been shown to stimulate osteoblast proliferation and alkaline phosphatase activity [27] and vitamin B_12 _deficiency has been associated with defective functional maturation of osteoblasts [28]. Recent publications indicate a shift to more evidence of osteoclast stimulation by high homocysteine and low vitamin B_12 _concentrations [29-31]. These mechanisms might be interrelated with another, with subsequent interference of homocysteine with collagen cross-linking. Cross-links are important for stability and strength of the collagen network. Interference in cross-link formation would cause an altered bone matrix, further resulting in more fragile bone [32].

Accordingly, these mechanistic studies support the hypothesis of a beneficial effect of homocysteine reduction by B-vitamin supplementation on fracture incidence and related outcome measures. However, it remains unknown whether this relationship is causal as evidence from Randomized Controlled Trials (RCTs) is still limited. It would be most valuable to assess this relationship in a population consisting of generally healthy elderly people as deficiencies of vitamin B_12 _and folate are highly prevalent in this population and lead to elevated homocysteine concentrations.

The primary aim of our current intervention is therefore to assess the efficacy of oral supplementation with vitamin B_12 _and folic acid in the prevention of fractures in Dutch elderly people with elevated homocysteine concentrations. We will address potential pathways and phenotypes leading to fractures, osteoporosis measures, falls and physical performance. We will concurrently address other outcomes associated with elevated homocysteine concentrations, such as cognitive function [33] and cardiovascular disease [34]. The aim of this article is to describe the design of our intervention and to describe the baseline characteristics of the population enrolled.

## Methods/Design

### Study design

The B-PROOF study is a randomized, placebo-controlled, double-blind, parallel intervention study. B-PROOF is an acronym for 'B-vitamins for the PRevention Of Osteoporotic Fractures'. This large multi-centre project is carried out in The Netherlands by a consortium from Erasmus MC (EMC, Rotterdam), VU University Medical Center (VUmc, Amsterdam) and Wageningen University (WU, Wageningen), the latter acting as coordinator. The study aimed to include 3000 subjects, aged 65 years and older, with elevated plasma homocysteine concentrations (≥ 12 μmol/L). The intervention period is 2 years. Participants were randomly allocated in a 1:1 ratio to the intervention group or to the control group. We stratified for study centre, sex, age (65-80 years, ≥ 80 years), and homocysteine concentration (12-18 μmol/L, ≥ 18 μmol/L). The intervention group receives a daily tablet with 500 μg vitamin B_12 _and 400 μg folic acid and the control group receives a daily placebo tablet. Both tablets contain 15 μg (600 IU) of vitamin D_3 _to ensure a normal vitamin D status [35]. The intervention and placebo tablets, produced by Orthica, Almere, the Netherlands, are indistinguishable in taste, smell and appearance. The random allocation sequence and randomization were generated and performed using SAS 9.2 by an independent research dietician.

Recruitment took place from August 2008 until March 2011. The B-PROOF study has been registered with the Netherlands Trial Register http://www.trialregister.nl under identifier NTR 1333 since June 1, 2008 and with ClinicalTrials.gov under identifier NCT00696514 since June 9, 2008. The WU Medical Ethics Committee approved the study protocol, and the Medical Ethics committees of EMC and VUmc gave approval for local feasibility.

### Sample size

Sample size calculation was based on the primary outcome measure of the intervention, i.e. osteoporotic fractures. The fracture rate in the non-treated group was estimated to be 5-6% in a period of two years, based on osteoporotic fracture incidence in both independently living and institutionalized elderly. Elderly in the highest quartile of homocysteine concentrations have been shown to have a doubled risk of fracture [10], we expected that the fracture rate in the treated group would be reduced by 34%. With a power of 80%, a significance level (α) of 0.05, one tail, 1500 participants were required for both intervention and placebo group. To compensate for the expected drop-out rate of 15%, we extended the intervention period with one year for the first 600 participants of the study.

### Subjects

Most participants were recruited via the registries of municipalities in the area of the research centres by inviting all inhabitants aged 65 years and older by mail. Furthermore, inhabitants of elderly homes in the area of Rotterdam, Amsterdam and Wageningen were invited to participate, after providing information brochures and information meetings. In addition, elderly who participated in previous studies of the research centres were approached. All participants gave written informed consent before the start of the intervention.

**Figure 1:**
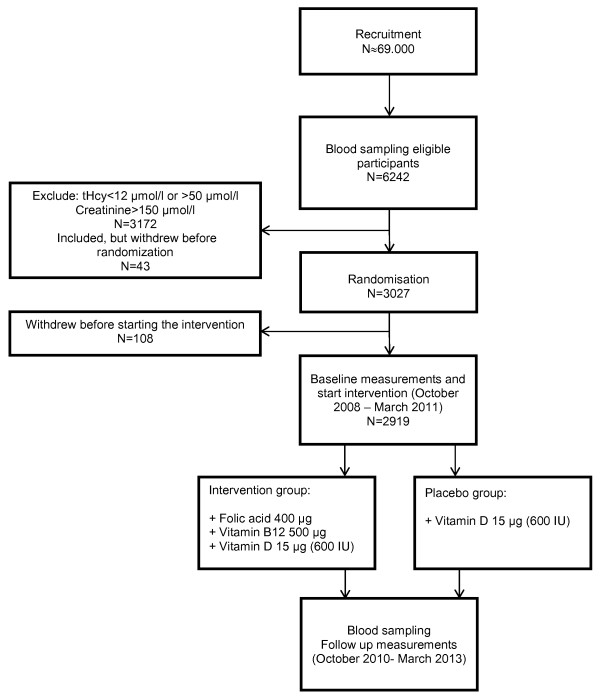
**Recruitment and baseline measurements in participants of the B-PROOF study**.

A total of 2919 subjects were included in the intervention (Figure 1). Inclusion and exclusion criteria are listed in Table 1.

**Table 1:** inclusion and exclusion criteria for the B-PROOF study

Inclusion criteria:	Exclusion criteria:
Men and women, aged 65 years and older	Immobilization: being bedridden or wheelchair bound
Compliance for tablet intake of > 85% 4-6 weeks prior to start of the trial	Cancer diagnosis within the last 5 year, except skin cancer as basal cell carcinoma and squamous cell carcinoma
Competent to make own decisions	Serum creatinine level > 150 μmol/L
Elevated homocysteine level (≥ 12 μmol/L and ≤ 50 μmol/L)	Current or recent (< 4 months) use of supplements with very high doses of vitamin B_12 _(intramuscular injections) or folic acid (> 300 μg)
	Participation in other intervention studies

### Changes to inclusion criteria after trial commencement

The inclusion criteria regarding cut-off values for plasma homocysteine concentrations and age were adapted during the first phase of the intervention. The initial eligibility criterion for plasma homocysteine concentrations has been adjusted from ≥ 15 μmol/L to ≥ 12 μmol/L before the start of the study. Extended data analyses (unpublished data), based on Van Meurs et al., 2004, showed that a relation between homocysteine status and fracture incidence is also present at a lower homocysteine concentration (~14 μmol/L). Furthermore, cross-calibration between different local homocysteine methods used in the current study (Architect Analyser, HPLC and LC-MS) and the methods used in the previous leading studies [9,10] showed that a homocysteine concentration of 14 μmol/L in these studies corresponded with a concentration of 12 μmol/L when using the current methods.

It was decided to adapt the criterion for age from 70 years and older to 65 years and older after the first year of recruitment, because the association between homocysteine and fractures is also present in people aged 65-70 years [9,10].

### Screening and run-in period

Blood samples were obtained from participants in the morning at the research centres or at an external location in the living area of the participants. Participants were in a fasted state, or had taken a light breakfast. Venous blood was drawn by a skilled nurse to obtain plasma, serum and buffy coats. For homocysteine analysis, a plasma EDTA tube was stored on ice immediately after blood drawing and samples were processed within 4 hours after blood drawing, to prevent a temperature- and time-dependent increase in plasma homocysteine [36]. Plasma homocysteine was measured using the Architect i2000 RS analyser (VUmc, intra assay CV = 2%, inter assay CV = 4%), HPLC method [37] (WU, intra assay CV = 3.1%, inter assay CV = 5.9%) and LC-MS/MS (EMC, CV = 3.1%). According to a cross-calibration, outcomes of the three centres did not differ significantly. Serum creatinine was measured with the enzymatic colorimetric Roche CREA plus assay (CV = 2%). The remaining plasma, serum and buffy coats samples were kept frozen at -80°C until further analysis.

After blood sampling participants started with a six-week run-in period, in which the participants took placebo tablets and were asked to daily fill out their study supplement intake on a research calendar. Subsequently, participants were informed whether they could further participate in the study or not, as an elevated plasma homocysteine concentration was an inclusion criterion, and an elevated serum creatinine concentration was an exclusion criterion. In case of laboratory results outside the reference range set for homocysteine (> 50 μmol/L) or creatinine (> 150 μmol/L) participants were referred to their general practitioner.

### Measurements

Eligible participants were invited for baseline measurements, which were performed during a 1.5-2 hour session at one of the study centres or at the participant's home. The 2-year intervention period started after these baseline measurements. Adherence was assessed by recordings on the research calendar, counts of bi-annually returned tablets, and periodical phone calls with the participants. After two years of intervention, participants are invited for follow-up measurements, in which the baseline measurements are repeated.

### Primary outcome

The primary outcome of the trial is time to first osteoporotic fracture. Participants recorded fractures on the research calendar, which was returned every 3 months. Incomplete or unclear data were further inquired by telephone. Furthermore, the research team verified reported fractures with the participants' general practitioner, hospital physician and/or by radiographs. All fractures are considered osteoporotic, except for head/hand/finger/foot/toe fractures and fractures caused by traffic accidents [38]. The time to fracture is the difference between starting date and date of fracture reported on the calendar or by the general practitioner.

### Secondary outcomes

#### Falls

Falls were recorded weekly on the research calendar. A fall was defined as an unintentional change in position resulting in coming to rest at a lower level or on the ground [39]. Recurrent falling was defined as at least two falls of a participant within six months during the two years of follow-up [40].

#### Dual Energy X-ray Assessment (DXA)

In two out of three study centres Dual Energy X-ray Assessment (DXA) was performed to measure bone mineral density (BMD) and lean body mass and to assess vertebral fractures, using the Hologic QDR 4500 Delphi device (VUmc, Hologic Inc., USA, CV = 0.45%) or the GE Lunar Prodigy device (EMC, GE Healthcare, USA, CV = 0.08%). The two devices were cross-calibrated. DXA was performed under standard protocols within four weeks after the participant's start of the intervention.

Total hip, femoral neck and lumbar spine BMD (g/cm^2^) were measured. Total hip BMD was measured at the left femur, while in case of a hip prosthesis at the left side, the right side was measured. Instant vertebral assessment (IVA) was performed to detect clinical and non-clinical vertebral fractures. Results were independently evaluated by two researchers, and inconsistencies were discussed.

Furthermore, total body composition was measured. The amount of fat-free soft tissue (i.e. lean mass minus bone mineral content) of the extremities can be used as an indicator of skeletal muscle mass and has been validated in older persons [41].

#### Quantitative Ultrasound (QUS)

Quantitative ultrasound (QUS) measurements of the calcaneus were performed using a Hologic Sahara bone densitometer (Hologic Inc., USA). Broadband ultrasound attenuation (BUA, dB/MHz, CV = 3.7%) and speed of sound (SOS, m/s, CV = 0.22%) were measured in duplicate in both the right and the left calcaneus. From these parameters, the quantitative ultrasound index (QUI, CV = 2.6%) and estimated BMD (eBMD) were calculated.

#### Bone turnover markers

After completion of the study, bone turnover markers will be determined in a subsample in order to obtain better insight in the mechanism underlying the effect of B-vitamin supplementation on bone health. Standard assays will be performed in baseline and follow-up blood samples to measure markers of bone formation and bone resorption, such as procollagen type 1 N-extension peptide (P1NP) and cross-linked carboxyterminal telopeptide of type 1 collagen (CTx).

#### Physical performance and handgrip strength

Physical performance was measured using three tests; a walking test, a chair stands test, and a balance test. These performance tests are commonly used in elderly people [42-44]. During the timed walking test, participants were asked to walk 3 meters, turn around, and walk back as quickly as possible. During the timed chair stands test the participants rose from and sat down in a chair as quickly as possible for five consecutive times without the use of their arms. Standing balance was assessed with the modified Romberg test in which the participants were asked to maintain balance for 10 seconds in four different positions with increasing difficulty. Each position was performed with eyes open and eyes closed.

Hand grip strength (kg) was measured using a strain-gauged dynamometer (Takei, TKK 5401, Takei Scientific Instruments Co. Ltd., Japan, inter observer CV = 5%). Participants were asked to perform two maximum hand grip trials with each hand in standing position with their arms along their body. Maximal hand grip strength was defined as the average of the highest score of the left and right hand.

#### Vascular parameters

Blood pressure measurements were performed using an Omron M1 plus blood pressure device (Omron Healthcare Europe). In two of the centres vascular structure and function was assessed non-invasively in a subsample by measuring blood pressure, intima-media-thickness (IMT) of the carotid artery, carotid distensibility (DC), aortic pulse wave velocity (PWV) and augmentation index (AIx).

Carotid B-mode ultrasonography is performed using the L105 40 mm 7.5 MHz array transducer (Picus, Pie Medical Equipment, Maastricht, the Netherlands) on the right carotid artery. IMT is evaluated as the distance luminal-intimal interference and the media-adventitial interface (Art.Lab, Esoate Europe, Maastricht, the Netherlands). The vessel wall movement-detector system has been described in detail previously [45]. The system consists of a wall track system and data-acquisition system (Art.Lab, Esoate Europe, Maastricht, the Netherlands). AIx is calculated using arterial tonometry obtained from the right radial, carotid and femoral artery using the Sphygmocor device (Sphygmocor version 7.1, AtCor Medical, Sydney, Australia). PWV is measured with simultaneously three channel ECG recording and recording of the right carotid and femoral artery pulse waveforms. Twenty-four hour ambulatory blood pressure recording was performed using Oscar 2 ambulatory 24 hour blood pressure monitor (SunTech Medical, North Carolina, USA).

#### Biomarkers of cardiovascular disease and cardiovascular events

Cardiovascular events were defined as cardiovascular mortality, myocardial infarction and stroke. Participants were requested to fill out a questionnaire regarding their cardiovascular history. After completion of the study cardiovascular and inflammatory biomarkers, such as amino-terminal B-type natriuretic peptide (NT-proBNP) and high-sensitivity hsC-reactive protein (hs-CRP) will be measured in baseline and follow-up blood samples.

#### Cognitive function

We used the Mini-Mental State Examination (MMSE) for a description of global cognitive performance in our study population [46]. In a subsample, i.e. all participants of WU, domain specific cognitive function was assessed using six standardized tests; the Symbol Digit Modalities Test, the Letter Fluency test, the Trail Making Test, the Digit Span Test, the Word Learning Test and the Stroop Colour Word Test. These tests were used to construct the following cognitive domains: attention, working memory, executive function, information processing speed and episodic memory [47].

#### Depression and Quality of Life

The Geriatric Depression Scale (GDS) was used to measure depressive symptoms [48]. To determine quality of life the EuroQoL EQ-5D [49] and Short Form Health Survey (SF-12) [50] questionnaires were used.

### Measurement of covariates

#### General self-reported health and medication usage

Self-reported medical history, ethnicity, use of medication and of nutritional supplements, current alcohol intake and smoking habits and history of falls and fractures were determined using a questionnaire.

Medication use during the study period was also retrieved from pharmacies. Data included the prescription period, the total amount of drug units per prescription, the prescribed daily number of units, product name, and the Anatomical Therapeutic Chemical (ATC) code.

#### Physical Activity

Physical activity was measured using the LASA Physical Activity Questionnaire (LAPAQ), which is a validated questionnaire to measure physical activity in elderly people [51]. The activities included walking, cycling, gardening, participation in sports and light and heavy household activities. Frequency and duration of each activity during the last two weeks were assessed. Physical activity was calculated in minutes/day and kcal/day.

#### Nutritional status and food intake

The Mini Nutritional Assessment (MNA) [52] and the Simplified Nutritional Appetite Questionnaire (SNAQ) [53] were used to screen for malnutrition and appetite loss. Standing height was measured in duplicate to the nearest 0.1 cm with the person standing erect and wearing no shoes. Weight was measured to the nearest 0.5 kg with the person wearing light garments without shoes and empty pockets. In a subsample, i.e. all participants of WU, we estimated dietary intake by a Food Frequency Questionnaire (FFQ) with its main focus on macronutrients, vitamin B_12_, folate, vitamin D, and calcium. The FFQ was developed by the dietetics group at the department of Human Nutrition, Wageningen University and was derived from an FFQ which was validated for energy, fat, cholesterol, folate and vitamin B_12 _intake [54,55].

#### Genotyping

From the blood samples drawn at baseline, DNA was isolated for genotyping. Subsequently, all samples were genotyped for approximately 700.000 single nucleotide polymorphisms (SNPs) using the Illumina Omni-express array, which has > 90% coverage of all common variation in the genome. If known functional SNPs were not tagged well by the array, they were genotyped separately using TaqMan allelic discrimination assays on the ABI Prism 9700 HT sequence detection system. The data will be used in a hypothesis-free genome-wide association study (GWAS) as well as in analyses of genetic variation in known candidate genes.

### Data analysis

The data analyses will be performed by following the intention-to-treat procedure (effectiveness study) and the per-protocol-procedure (efficacy study). If necessary, data will be transformed and analyses will be adjusted for the presence of covariates. Time to first fracture will be analysed using Cox Proportional Hazard Models.

Differences in mean change between groups will be analysed with independent sample Student's t-test, ANOVA or other similar tests.

Two-sided P values will be calculated and a significance level of 0.05 will be applied.

We did not perform an interim analysis because we did not expect and observe negative side effects of the supplementation and because of the relatively long recruitment period, with most of the participants included in the last year of recruitment. We keep track of any serious adverse events (SAEs) occurring during the duration of the study.

### Inclusion and baseline characteristics of the participants

Baseline characteristics of participants in the B-PROOF study are shown in Table 2. During the recruitment, we addressed approximately 69.000 people (Figure 1). This resulted in the screening of 6242 interested persons, of which 3027 were eligible to participate. One hundred and eight participants withdrew consent before start of the intervention resulting in 2919 participants who completed baseline measurements. The mean age of participants at the start of the intervention was 74.1 years (SD: 6.5) and 50% was female. Median plasma homocysteine concentration was 14.1 μmol/L (IQR: 13.0-16.6).

**Table 2:** Baseline characteristics of the B-PROOF study participants

	Total (n = 2919)	Male (n = 1456)	Female (n = 1463)
Study location (n)			
-WU	856	499	357
-VUmc	778	301	477
-Erasmus MC	1285	656	629

Age (years)*	74.1 (6.5)	73.4 (6.1)	74.9 (6.8)

Plasma homocysteine (μmol/L)^#^	14.4	14.6	14.1
	[13.0-16.6]	[13.1-16.8]	[12.9-16.3]

Serum creatinine (μmol/L)^#^	82.0	90.0	73.0
	[71-94]	[81.0-101.0]	[65.0-84.0]

Weight (kg)^#^	77.9 (13.3)	83.1 (11.9)	72.7 (12.5)

Height (cm) ^#^	169.3 (9.3)	175.9 (6.6)	162.7 (6.6)

Physical activity (min/day)^#^	130.0	116.3	142.9
	[84.0-192.9]	[72.5-177.0]	[96.0-205.7]

Years of education*	10.1 (4.0)	10.9 (4.1)	9.2 (3.6)

Smoking (%)			
- Current	9.6	10.8	8.5
- Former	56.5	69.1	44.0
- Never	33.9	20.1	47.6

*Results are presented in mean (standard deviation); ^#^Results are presented in median [interquartile range].

## Discussion

To our best knowledge, the B-PROOF study is the first intervention study in which the effect of vitamin B_12 _and folic acid supplementation on osteoporotic fractures is studied in a general elderly population. Currently, folic acid fortification is not mandatory in the Netherlands, and it is only applied on small scale in bread substitutes. This intervention is therefore an excellent opportunity to investigate the effect of folic acid and vitamin B_12 _supplementation in a non-fortified population. Positive evidence emerging from this intervention might enable elderly to live into an advanced age with lower fracture risk. Implementation of vitamin B_12 _and folic acid supplementation might therefore reduce the costs of national health services for osteoporosis in the elderly.

Elevated homocysteine concentrations are associated with various health outcomes, but until now there are no large interventions investigating the effect of homocysteine lowering treatment on, for example, physical performance. Therefore, the wide range of secondary outcomes studied in the B-PROOF study is unique. The possibility to perform a GWAS in such a large general elderly population will provide us with relevant data on the underlying mechanisms and genes involved in age-related diseases as osteoporosis and cognitive decline. In addition, DNA analysis gives us the opportunity to focus on the effect of B-vitamins on epigenetic changes.

We have some remarks on the expected outcomes of this study. We expect the effect of folic acid and vitamin B_12 _supplementation to be most beneficial in people with an elevated homocysteine concentration. We therefore only included elderly people with elevated homocysteine concentrations (≥ 12 μmol/L), but as a consequence, we cannot extrapolate the results to elderly with low to normal homocysteine concentrations (< 12 μmol/L). However, 49% of the elderly screened in our study had an elevated homocysteine concentration. This percentage might be higher in the general Dutch elderly population, since people interested in nutrition and health, with a subsequent healthier lifestyle are probably more willing to participate in a long term intervention study. Therefore, the B-PROOF study covers a large segment of the general Dutch elderly population.

Because we supply both folic acid and vitamin B_12_, it will not be possible to indicate whether the effects of the intervention will be the consequence of folic acid or vitamin B_12 _supplementation or lowering homocysteine concentrations in general. However, since both vitamins play a significant role in homocysteine metabolism, and folic acid supplementation alone might mask a possible vitamin B_12 _deficiency [56], it is the most efficient and safest to supplement both vitamins.

The first longitudinal results of the B-PROOF study will become available in the second semester of 2013.

## Competing interests

The B-PROOF study has received funding so far from NZO (Dutch Dairy Association), Zoetermeer, and Orthica, Almere, the Netherlands. The sponsors have no role in the design or implementation of the study, data collection, data management, data analysis, data interpretation, or in the preparation, review, or approval of the manuscript.

## Authors' contributions

JPVW, EMBB, KMAS, AWE, SCVD implement the practical realisation of the study.

RAMDR, LCPGMDG and PL designed and initiated the trial. LCPGMDG is the principal investigator. LCPGMDG, PL and AGU represent the scientific committee of the B-PROOF study. RAMDR is the overall trial coordinator and NMVS and NVDV are local trial coordinators. RAMDR, NMVS, NVDV, JPVW, AWE, SCVD, KMAS, MCZ, JBJVM and JB planned and coordinated the study, JPVW, AWE, SCVD, KMAS and EMBB are responsible for data collection and management and perform statistical analyses, interpret results, JPVW drafted the manuscript. All authors assisted in interpretation of the results, critically reviewed the manuscript, and approved the final draft.

## Pre-publication history

The pre-publication history for this paper can be accessed here:

http://www.biomedcentral.com/1471-2318/11/80/prepub
